# Immunoinformatics-guided approach for designing a pan-proteome multi-epitope subunit vaccine against African swine fever virus

**DOI:** 10.1038/s41598-023-51005-3

**Published:** 2024-01-16

**Authors:** Alea Maurice Simbulan, Edward C. Banico, Ella Mae Joy S. Sira, Nyzar Mabeth O. Odchimar, Fredmoore L. Orosco

**Affiliations:** 1 Department of Science and Technology, Virology and Vaccine Research and Development Program, Industrial Technology Development Institute, Bicutan, 1634 Taguig, Metro Manila Philippines; 2 Department of Science and Technology, S&T Fellows Program, Bicutan, 1634 Taguig, Metro Manila Philippines; 3 Department of Biology, University of the Philippines Manila, 1000 Manila, Philippines

**Keywords:** Protein analysis, Protein design, Protein structure predictions, Proteome informatics

## Abstract

Despite being identified over a hundred years ago, there is still no commercially available vaccine for the highly contagious and deadly African swine fever virus (ASFV). This study used immunoinformatics for the rapid and inexpensive designing of a safe and effective multi-epitope subunit vaccine for ASFV. A total of 18,858 proteins from 100 well-annotated ASFV proteomes were screened using various computational tools to identify potential epitopes, or peptides capable of triggering an immune response in swine. Proteins from genotypes I and II were prioritized for their involvement in the recent global ASFV outbreaks. The screened epitopes exhibited promising qualities that positioned them as effective components of the ASFV vaccine. They demonstrated antigenicity, immunogenicity, and cytokine-inducing properties indicating their ability to induce potent immune responses. They have strong binding affinities to multiple swine allele receptors suggesting a high likelihood of yielding more amplified responses. Moreover, they were non-allergenic and non-toxic, a crucial prerequisite for ensuring safety and minimizing any potential adverse effects when the vaccine is processed within the host. Integrated with an immunogenic 50S ribosomal protein adjuvant and linkers, the epitopes formed a 364-amino acid multi-epitope subunit vaccine. The ASFV vaccine construct exhibited notable immunogenicity in immune simulation and molecular docking analyses, and stable profiles in secondary and tertiary structure assessments. Moreover, this study designed an optimized codon for efficient translation of the ASFV vaccine construct into the *Escherichia coli* K-12 expression system using the pET28a(+) vector. Overall, both sequence and structural evaluations suggested the potential of the ASFV vaccine construct as a candidate for controlling and eradicating outbreaks caused by the pathogen.

## Introduction

African swine fever virus (ASFV) is the causative agent of African swine fever (ASF), a highly contagious and devastating viral disease that causes hemorrhagic fever and death among domestic and wild pigs, warthogs, and bushpigs^1^. As of June 2023, this disease is classified as a notifiable disease with reports in 50 countries since 2021 and has led to nearly 4000 outbreaks in domestic pigs and 17,000 outbreaks in wild boars, resulting in a loss of approximately 1.5 million swine^2^. Although non-zoonotic, it poses an ongoing threat as it continuously devastates the pork industry. China, the largest global exporter of pork, suffered a direct economic loss of US$ 141 billion in 2019, only a year after the emergence of ASF^3^.

The absence of globally approved and licensed vaccines for ASFV in commercial pig populations underscores the need for its development. There are three main strategies for the development of ASFV vaccine candidates^4,5^. Whole inactivated vaccines have proven ineffective in inducing antibodies and cytotoxic T-lymphocytes (CTLs), which are crucial for virus-infected cell elimination^6–8^. A live attenuated virus vaccine from Vietnam had shown efficiency^9^, yet additional safety and cross-protective ability tests must be performed. Subunit vaccines, a safer and cheaper alternative to live attenuated vaccines, use purified recombinant proteins or synthetic peptides that are capable of inducing protective immune responses^10^. However, in contrast to vaccines derived from inactivated or live-attenuated pathogens, subunit vaccines exhibit limited immunogenicity^11,12^. Therefore, the addition of adjuvants becomes essential to increase their efficiency, facilitating the induction of stronger and long-lasting protective immune responses^13^.

Numerous publications have discussed the diverse efforts in the development of a subunit vaccine for ASFV^5,14–17^. Most vaccine designs have involved the use of entire proteins^18,19^ or combinations of multiple proteins^20–23^. While many of these designs demonstrated efficacy against challenges of homologous strains^19,20^, a significantly lowered protection conferred after a challenge of heterologous strains was also reported^24^. This established an observation of a strain-specific immune response generated by ASFV subunit vaccines. To solve this, screening of antigenic components should involve tests for conservation across multiple strains. This increases the efficiency of the subunit vaccine design by enhancing its genotype coverage^25^.

Current research is focused on identifying short and specific antigenic regions called epitopes as vaccine components, as large proteins usually trigger allergic reactions^26^. Vaccination of epitope components has been proven to prolong the survival of domestic pigs following infection^27^. The administration of combinations of epitopes derived from p220, p72, and p30 resulted in a significant delay in the mortality of infected pigs by four days^27^. The efficacy of this design could potentially be optimized by identifying the right combination of epitopes. Currently, the most suitable epitope combination for a vaccine remains unidentified. Therefore, there is a need to explore and test different epitope combinations in challenge studies.

Mapping of epitopes within ASFV poses a great challenge due to its large genome and complex nature, encoding over 150 open reading frames (ORFs). The majority of these ORFs lack information on expression and functions^14^. One promising approach in epitope-based vaccine development is immunoinformatics, which utilizes bioinformatics to gain insights into immune system responses. Various computational tools enable the mapping and assessment of potential epitopes within the pathogen sequence.

This study aimed to unbiasedly search for potential epitopes that can be recognized by the swine immune system through pan-proteomic screening. In contrast to previous *in silico* studies that concentrated solely on structural proteins^28–32^, screening of epitopes in this study covered the entire ASFV proteome including nonstructural proteins and those that have not been characterized. Some nonstructural and uncharacterized proteins of ASFV have been found to function as antigens and immunogens. These proteins have been recognized by infected pig sera and some were observed to induce both T-cell and antibody responses^15^. This suggests their potential as targets for vaccine development. Moreover, sequence and structure-guided evaluations of the vaccine construct were performed to ensure its safety, stability, and immunogenicity against pathogenic ASFV.

## Results

### Proteome screening and protein prioritization

The study retrieved 197 distinct proteomes of the African swine fever virus (ASFV) from NCBI and UniProt. Only 100 well-annotated ASFV proteomes were retained following the exclusion of 21 proteomes with fewer proteins than the standard 150^33^, six (6) proteomes with partial genome coverage, and 70 proteomes with either unverified tags from NCBI or from the excluded section of UniProt^34^. With only 33 proteomes having available genotype assignments from NCBI, the genotypes of the remaining 67 proteomes were determined through phylogenetic-based sequence clustering analysis using 33 proteomes with genotype assignments as reference sequences. The list of accession numbers of the final proteomes considered in the study is in Supplementary Table 1 while their distribution profiles are presented in Supplementary Fig. 1.

The final ASFV proteomes have 18,858 proteins where 2,978 were identified as nonredundant by CD-HIT. Only 163 proteins from all the nonredundant proteins were found in at least 80% of genotype I or 80% of genotype II proteomes. From 163 protein sequences, 93.80% of the amino acid residues were identified as conserved by Protein Variability Server (PVS). For the prediction of cytotoxic T-lymphocyte (CTL) epitopes, 532 peptides with $\ge$9 consecutively conserved amino acids were selected, whereas for helper T-lymphocyte (HTL), 450 peptides with $\ge$15 consecutively conserved amino acids were selected.

### Epitope prediction

From the conserved fragments of 163 protein sequences, 83 peptides were predicted as possible linear B-lymphocyte (LBL) epitopes by four LBL prediction servers (BepiPred 3.0, ABCPred, SVMTrip, and LBTope). From 532 peptides with $\ge$9 consecutively conserved amino acids, 47,165 9-mer peptides were identified as possible cytotoxic T-lymphocyte (CTL) epitopes by IEDB MHCI-binding prediction server, whereas from 450 peptides with $\ge$15 consecutively conserved amino acids, 44,773 15-mer peptides were identified as possible helper T-lymphocyte (HTL) epitopes by IEDB MHCII-binding prediction server. The screening process for each epitope group (LBL, CTL, and HTL) is presented in Supplementary Fig. 2. The final list of epitopes and their protein sources is shown in Table 1.

**Table 1:** Final epitope components of the African swine fever virus (ASFV) vaccine construct, and their respective protein sources.

Groups	Epitopes	Source proteins (ORFs)
LBL	GIAGRGIPLGNPHVKP	Minor capsid protein p49 (B438L)
	LDAVKMDKRNIK	Envelope protein p22 (KP177R)
	AKLQDTKFKWKYTLDP	Transmembrane protein (C257L)
	GRPSRRNIRFK	Major capsid protein p72 (B646L)
CTL	TVSAIELEY	Polyprotein p220 (CP2475L)
	KTRDFFILY	Transmembrane (C62L)
	MMDFERVHY	Cysteine protease (S273R)
	KNLSIIWEY	MGF 360-13L
	KAIELYWVF	MGF 360-18R
	YLYEIEIEY	Guanylyltransferase (NP868R)
HTL	SRRFRFVSLDAYNMG	Termination factor (Q706L)
	KIGFYSSKSTAHERE	Helicase/primase (F1055L)
	ESVYFAVETIHLKQQ	EP424R
	KYWYAIAVDYDLKDA	MGF 360-11L

All screened epitopes were non-allergens, non-toxins, and had antigenicity values >1.0. Only these properties were considered in the screening of LBL epitopes. The antigenicity scores of LBL epitopes are displayed in Table 2.

**Table 2:** Antigenicity scores of final linear B-lymphocyte (LBL) epitopes.

CTL epitopes	Antigenicity Scores
GIAGRGIPLGNPHVKP	1.43
LDAVKMDKRNIK	1.03
AKLQDTKFKWKYTLDP	1.37
GRPSRRNIRFK	1.83

Aside from non-allergens, non-toxins, and antigenicity >1.0, the final CTL epitopes have immunogenicity >0.25, proteasomal cleavage and TAP scores >1.0, and strong binding affinity to $\ge$9 swine leukocyte antigens (SLA). The scores of final CTL epitopes on antigen processing and presentation servers are presented in Table 3.

**Table 3:** Scores of final cytotoxic T-lymphocyte (CTL) epitopes on antigen processing and presentation servers.

CTL epitopes	Antigenicity	No. of strong	Cleavage	TAP	Immunogenicity
	Scores	binding MHCI	Scores	Scores	Scores
TVSAIELEY	1.71	19	1.21	1.39	0.26
KTRDFFILY	1.49	24	1.45	1.33	0.37
MMDFERVHY	1.44	17	1.54	1.29	0.32
KNLSIIWEY	1.41	20	1.51	1.28	0.33
KAIELYWVF	1.36	24	1.53	1.26	0.34
YLYEIEIEY	1.09	26	1.30	1.28	0.49

While final HTL epitopes were also non-allergens, and non-toxins, and had antigenicity scores >1.0, they also have immunogenicity >20 and strong binding affinity to $\ge$6 human leukocyte antigens (HLA). Some epitopes were also inducers of interferon-$\gamma$ (IFN-$\gamma$), interleukin-4 (IL-4), and interleukin-10 (IL-10). The scores of final HTL epitopes on antigen presentation and cytokine-inducing prediction servers are presented in Table 4.

**Table 4:** Scores of final helper T-lymphocyte (HTL) epitopes on MHCII antigen presentation and cytokine-inducing prediction servers.

HTL epitopes	Antigenicity scores	No. of strong binding MHCII	Immunogenicity scores	IFN-$\gamma$ inducer	IL-4 inducer	IL-10 inducer
SRRFRFVSLDAYNMG	1.40	14	36.51	No	Yes	Yes
KIGFYSSKSTAHERE	1.33	6	34.96	No	No	Yes
ESVYFAVETIHLKQQ	1.12	13	44.07	No	Yes	Yes
KYWYAIAVDYDLKDA	1.01	7	20.41	Yes	Yes	Yes

### Vaccine construction and evaluation

**Figure 1:**
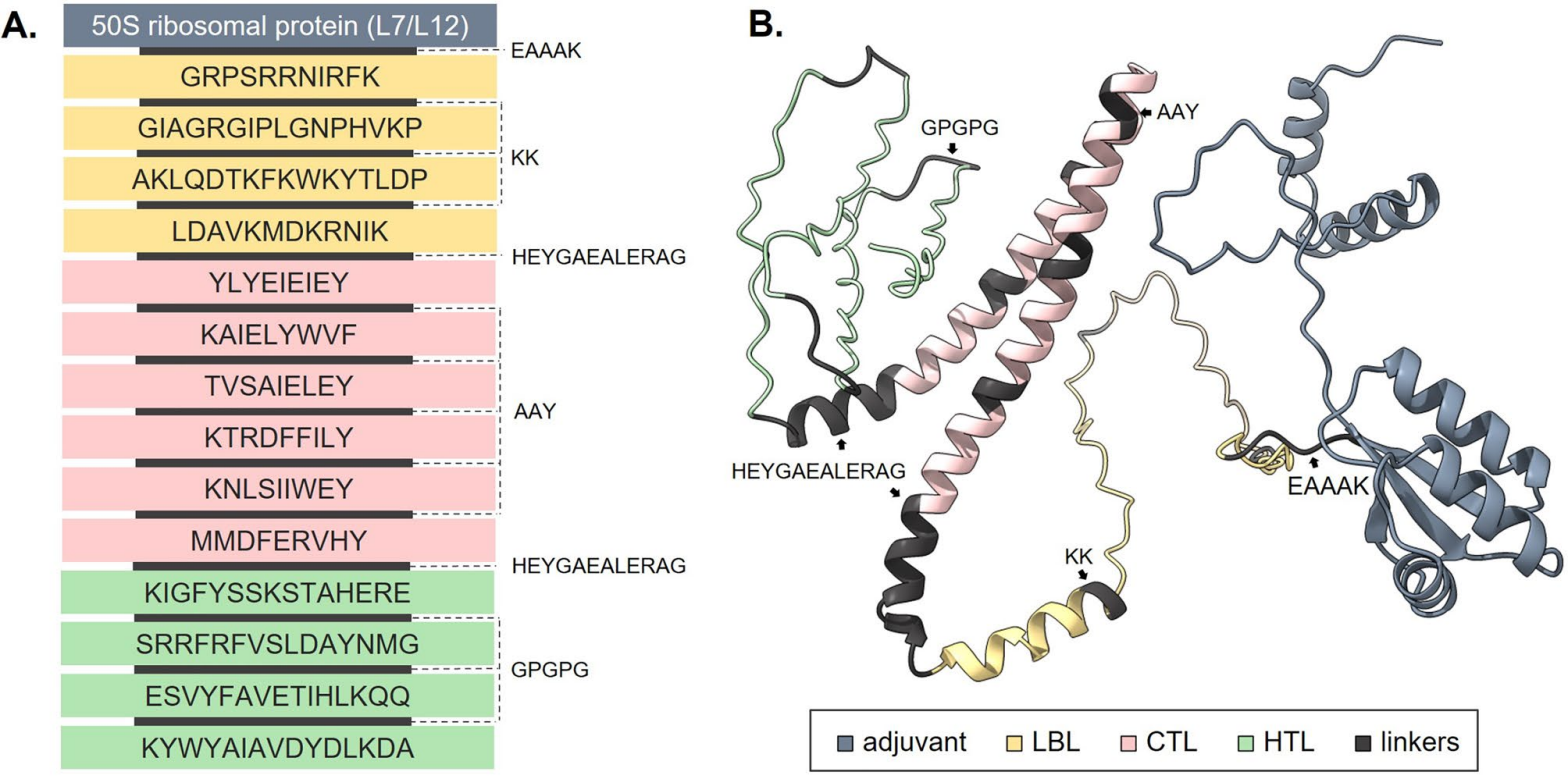
Designed multi-epitope subunit vaccine for African Swine Fever Virus (ASFV). (**A**) Graphical presentation showing the positions of epitopes, adjuvant, and linkers. (**B**) Tertiary model predicted by ColabFold v1.5.2, refined by GalaxyRefine, and visualized by ChimeraX.

“KK”, “AAY”, and “GPGPG” linkers were used to connect LBL, CTL, and HTL epitopes, respectively. “HEYGAEALERAG” linker was used to connect these three epitope groups. Five (5) vaccine candidates were prepared by adding the sequences of five different adjuvants (*Sus scrofa* $\beta$-defensin-1, F3-A6 ASFV hemagglutinin peptides, phenol-soluble modulin $\alpha$4, 50S ribosomal protein L7/L12, and heparin-binding hemagglutinin adhesin) to the epitopes using “EAAAK” peptide. The five vaccine candidates were subjected to physicochemical property evaluations (See Supplementary Table 2). All of the vaccine candidates were non-allergens and had antigenicity scores >0.4. However, structure instability was observed among the vaccine candidates with *Sus scrofa* $\beta$-defensin-1, F3-A6 ASFV hemagglutinin peptides, and heparin-binding hemagglutinin adhesin adjuvants. Insolubility was observed from the vaccine candidate with phenol-soluble modulin $\alpha$4 adjuvant. Therefore, 50S ribosomal protein L7/L12 was chosen as the most suitable adjuvant for the vaccine construct. Table 5 displays the complete physicochemical properties of the final ASFV vaccine construct using the 50S ribosomal protein L7/L12 as an adjuvant. Overall, the vaccine construct has 364 amino acids. Figure 1A illustrates the arrangement of the adjuvant, epitopes, and joining linkers.

**Table 5:** Complete physicochemical properties of the final vaccine construct and the servers used for evaluations.

Servers	Properties	Results
Protparam	Molecular weight	40,209.06
	Isoelectric point	7.04
	Stability	Stable (instability index = 32.5)
	Aliphatic index	82.47
VaxiJen 2.0	Antigenicity	Antigenic (0.76)
AllerTOP 2.0	Allergenicity	Non-allergenic
BlastP	Cross-reactivity	Zero
SolPro	Solubility	Soluble (87.6% probability)

Secondary structure characterization by GOR4 revealed that the vaccine construct has 58.38% residues in $\alpha$-helices, 29.19% in random coils, and 12.43% in extended strands. Figure 1B shows the tertiary model of the ASFV vaccine construct predicted by ColabFold v1.5.2, refined by GalaxyRefine, and visualized by ChimeraX.

The ASFV vaccine construct achieved the recommended scores for high-quality structures in three quality assessment servers (see Supplementary Fig. 3) with a z-score of −3.73 in ProSA and an overall quality factor of 96.4% in ERRAT while 97.5% of its residues are in favorable regions as predicted by PROCHECK.

GlobPlot2 identified two (2) globular regions in the ASFV vaccine construct: M1-I150 and K165-K297, and four (4) disordered regions: A151-P164, S298-R312, D319-S331, and Q343-K350. The first globular region spanned the entire adjuvant region and the first LBL epitope while the second globular region approximately covered the 3rd–4th LBL epitopes and all CTL epitopes. Predicted disordered regions spanned the 2nd LBL epitope (GIAGRGIPLGNPHVKP) and segments of HTL epitopes. Discotope and Ellipro identified eight (8) conformational B-lymphocyte epitopes within the multi-epitope region: R144-K146, K148-P164, K166-Q170, T179-E205, T299, H301-R311, L341-K350, and K362-A364. No conformational B-lymphocyte epitope was predicted within the adjuvant. The predicted globular-disordered regions and conformational B-lymphocyte epitope regions within the ASFV vaccine construct are displayed in Supplementary Fig. 4.

### Immune simulation

**Figure 2:**
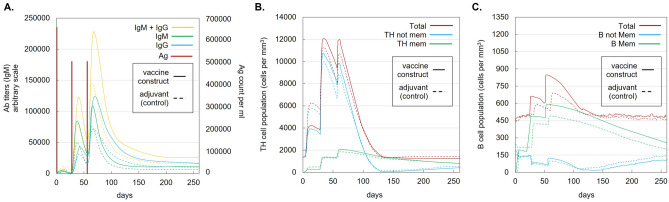
In silico immune simulation results of the ASFV vaccine construct (solid lines) compared with adjuvant alone (dash line) administered at 0-4th-8th week intervals. Graphs: (**A**) immunoglobulin production and antigen clearance. (**B**) Helper T-lymphocyte populations. (**C**) B-lymphocyte populations.

Immune simulations using C-ImmSim revealed increasing amounts of antibody titers and population counts of helper T-lymphocytes (HTL) and B-lymphocytes from primary to secondary and tertiary immune responses. A decrease in antigen count was also observed after secondary administration of the vaccine construct. Figure 2 compares the immune profiles of the ASFV vaccine construct to the adjuvant (50S ribosomal protein L7/L12) alone. The ASFV vaccine construct has relatively higher antibody titers and HTL and B-lymphocyte populations compared to the adjuvant alone.

### Molecular docking and molecular dynamics

The CTL epitopes predicted in this study were docked to an MHCI molecule, SLA-1*04:01. The crystal structure of SLA-1*04:01 was retrieved from RCSB ID: 3QQ3 after the removal of an influenza-derived epitope (NSDTVGWSW) that was originally complexed in the structure. This epitope was used as a positive control in the study together with an ebola-derived epitope (ATAAATEAY) complexed to SLA1*0401 in another crystal structure (RCSB ID: 3QQ4).

The ASFV vaccine construct was also docked to a crystal structure of toll-like receptor 4 (TLR4) from RCSB ID: 4G8A. For positive control, three (3) TLR4 protein agonists: enzyme lumazine synthase from *Brucella* spp., resuscitation-promoting factor (Rpf) E from *Mycobacterium tuberculosis*, and fusion protein DnaJ-$\Delta$A146Ply from *Streptococcus pneumoniae* were used. The structures of the complexes predicted by Cluspro 2.0 are shown in Supplementary Fig. 5. The figures indicate the residues and specific atoms that bind or contact with TLR4. The predicted number of molecular interactions and binding energies of SLA-1*04:01 and TLR4 complexes are displayed in Table 6.

**Table 6:** Predicted molecular interactions and binding energies ($\Delta$G) of SLA-1*04:01 and TLR4 complexes.

Complex	Molecular interactions	$\Delta$G (kcal/mol)
SLA1*04:01-TVSAIELEY	13 HB, 130 NBC	−12.1
SLA1*04:01-KTRDFFILY	11 HB, 142 NBC	−12.1
SLA1*04:01-MMDFERVHY	9 HB, 108 NBC	−11.3
SLA1*04:01-KNLSIIWEY	12 HB, 156 NBC	−11.8
SLA1*04:01-KAIELYWVF	8 HB, 116 NBC	−11.6
SLA1*04:01-YLYEIEIEY	10 HB, 129 NBC	−11.3
SLA1*04:01-NSDTVGWSW$^{\rm a}$	11 HB, 120 NBC	−10.8
SLA1*04:01-ATAAATEAY$^{ \rm b}$	14 HB, 142 NBC	−11.2
TLR4-ASFV construct	6 SB, 15 HB, 199 NBC	−14.0
TLR4-*Brucella* lumazine synthase$^{\rm c}$	7 SB, 15 HB, 257 NBC	−9.9
TLR4-*M. tuberculosis* RpfE$^{\rm d}$	6 SB, 31 HB, 262 NBC	−9.7
TLR4-*S. pneumoniae* DnaJ$^{\rm e}$	4 SB, 18 HB, 156 NBC	−13.1

*HB* hydrogen bonds, *NBC* non-bonded contacts, *SB* salt bridges.

Control: $^{\rm a}$influenza-derived epitope from RCSB ID: 3QQ3 and $^{\rm b}$ebola-derived epitope from RCSB ID: 3QQ4, both bound to SLA1*0401 in crystal structures. $^{\rm{c-e}}$TLR4-agonists.

Although the “ATAAATEAY” control has the highest number of hydrogen bonds, two of the CTL epitopes in the study (“TVSAIELEY” and “KNLSIIWEY”) have higher numbers of hydrogen bonds compared to the “NSDTVGWSW” control. Despite the lower number of molecular interactions observed, all CTL epitopes of the vaccine construct displayed higher $\Delta$G (kcal/mol) compared to the controls.

The ASFV vaccine construct also exhibited lower numbers of molecular interactions in complex with TLR4 but displayed the highest $\Delta$G (kcal/mol) compared to the controls. While in molecular dynamics simulation of the TLR4 complexes, the ASFV vaccine construct had also the lowest eigenvalue with 4.85E−07, followed by *S. pneumoniae* DnaJ (2.69E−06), *M. tuberculosis* RpfE (1.46E−05), and *Brucella* lumazine synthase (1.57E−05). The graphs for main-chain deformability, B-factor, eigenvalues, variances, covariance, and elastic network are shown in Supplementary Fig. 6.

### Codon optimization and in silico cloning

**Figure 3:**
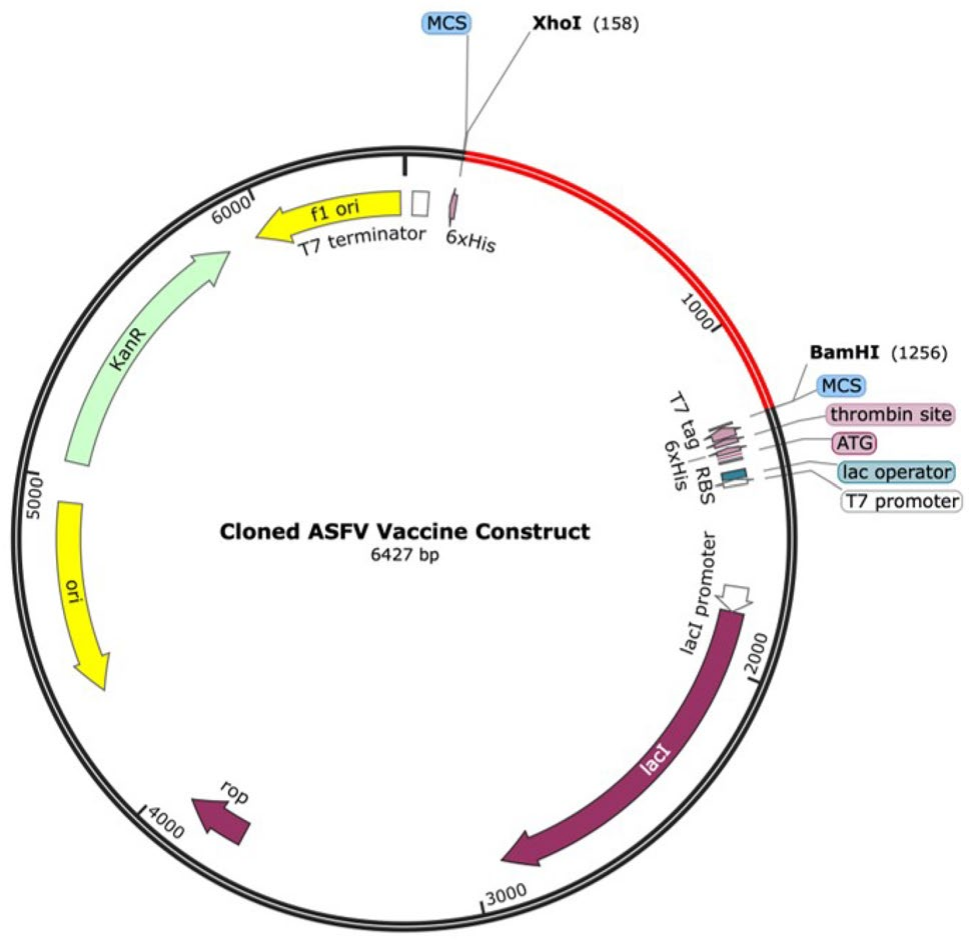
Result of in silico restriction cloning of the ASFV vaccine construct into the pET28a(+) vector using SnapGene software. The adapted sequence was inserted at XhoI (158) and BamHI (1256) restriction sites and is indicated in red, while the plasmid backbone was kept in black.

Following optimization of the codon sequence of the vaccine construct in expression to *Escherichia coli* K-12 expression system, the codon adaptation index (CAI) predicted a value of 1.0 and a GC content of 48.26%. Sample cloning of the adapted sequence of the vaccine construct into the pET28a(+) vector is shown in Fig. 3. The adapted sequence was inserted into XhoI (158) and BamHI (1256) restriction sites.

## Discussion

The landscape of vaccine development has undergone significant transformations due to discoveries in immunology. Immunoinformatics, an approach that combines immunology with bioinformatics, led to the emergence of a new pattern of vaccine design where immune determinants can simply be scanned in the protein sequence of the pathogen of interest. Compared to conventional vaccine design, which makes use of extensive wet laboratory experiments, immunoinformatics-based vaccine design drastically reduces both time and labor needs in epitope screening. The shortened process of developing vaccines that this approach offers can effectively address the rapid emergence or re-emergence of numerous highly pathogenic infectious diseases.

This study used immunoinformatics to map epitopes and design a multi-epitope subunit vaccine for the African Swine Fever virus (ASFV), the causative agent of the highly contagious and fatal African Swine Fever (ASF) disease. Moreover, pan-proteomic screening was used to identify immunogenic epitopes, not only within structural proteins but even in the nonstructural proteins of ASFV. The inclusion of nonstructural viral proteins in this study was influenced by recommendations from various studies on different viruses citing that numerous nonstructural proteins are involved in viral activity and can also trigger host immunity^35,36^. ASFV genome encodes over 100 non-structural proteins^37^, in which the functions of many of these proteins remain unknown. While a previous vaccine design study was able to screen T-cell epitopes on nonstructural proteins of ASFV^38^, it is important to note that nonstructural proteins of ASFV can also elicit antibody responses to swine hosts^15^, indicating their significance in the prediction of B-cell epitopes. B-cell epitopes from nonstructural proteins have also been included in the designs of multi-epitope subunit vaccines targeting other pathogens^35,39,40^.

In screening for potential epitopes, this study prioritized proteins found in genotypes I and II. Isolates of ASFV can be classified into 24 genotypes^41^; however, only genotypes I and II have caused economically devastating epidemics outside Africa^42^. Previous vaccine design studies^43,44^ have recognized the importance of identifying epitopes from these genotypes, specifically genotype II^38^, the genotype responsible for the 2007-2022 ASF outbreaks^45^.

Four (4) linear B-lymphocyte (LBL), six (6) cytotoxic T-lymphocyte (CTL), and four (4) helper T-lymphocyte (HTL) epitopes were considered as components for the multi-epitope subunit vaccine for ASFV. These epitopes were non-allergenic, non-toxic, and highly antigenic. Non-allergenicity and non-toxicity assessments are crucial to ensure safety when the vaccine is processed within hosts. Moreover, all epitopes demonstrated antigenicity that surpassed the score of 1.0, the threshold set for highly antigenic epitopes^46^. Therefore, these epitopes are expected to have a substantial ability to be recognized as foreign materials that can potentially trigger an immune response.

The utilization of four prediction servers ensured the accuracy of the predicted LBL epitopes. The decision to prioritize BepiPred 3.0 server for the prediction was influenced by a study in which the server demonstrated its superior accuracy compared to other LBL epitope prediction servers^47^. Moreover, utilizing SVMTrip, ABCPred, and LBTope increased the confidence in the predicted epitopes. The combination of multiple servers in LBL prediction was adapted in a previous study^48^, however, the current study opted for LBTope over BCEPred, as LBTope demonstrated higher accuracy performance in a recent benchmark study^47^.

To predict CTL epitopes, this study utilized tools spanning various stages of antigen presentation. These stages encompass proteasomal cleavage, TAP transport, binding to MHC-I molecules, and presentation of peptides to T-cell receptors. The NetCTLpan server covered the initial three stages. Although NetCTLpan predicts MHC-I binding, the study employed a separate server dedicated solely to MHC-I binding, NetMHCpan, owing to its extensive training on larger datasets^49^. Additionally, an immunogenicity server was employed to predict the relative capability of a given set of peptides bound within an MHC complex to be recognized by T-cell receptors. Altogether, the integration of these prediction tools in this study ensured that epitopes will be presented accurately to T-cells, and consequently induce an immune response.

To validate the result of the MHCI binding predictions, molecular docking of the final CTL epitopes to an MHCI molecule, SLA-1*04:01, was performed. This MHCI molecule was prevalent in different swine breeds^50^, and was already used for docking with CTL epitopes in other swine vaccine design studies^28^. Two (2) known CTL epitopes bound with SLA-1*04:01 in crystal structures^51^ were used as controls in the study. The docking results revealed highly negative Gibbs Free energies ($\Delta$G) for the CTL epitopes, with some epitopes displaying higher numbers of molecular interactions compared to the control group. This observation strongly implies the formation of stronger bound complexes for these epitopes and the SLA molecule. This study primarily employed a single MHCI molecule for CTL epitope docking due to the constraints posed by the limited availability of MHCI molecules in existing databases. However, it is recommended to expand the scope of this investigation by including a broader range of MHCI molecules in future docking studies. Polymorphisms in MHCI molecules affects the binding specificity in which the amino acids interact within the pockets of MHCI molecules^52^. Since the anchor residues for peptide binding are different for each allelic variant, diversification in the MHCI molecules used for docking analysis is a critical aspect to be considered.

In the prediction of HTL epitopes, only binding and immunogenicity servers were available. In this study, two binding prediction servers were used to predict the binding affinity of peptides to MHCII molecules. Multiple servers can be used for cross-validation of the results which can enhance the accuracy, reliability, and depth of epitope prediction. Unlike MHCI binding predictions, human leukocyte antigens (HLAs) were used for MHCII binding predictions since no swine leukocyte antigens (SLAs) were available in the servers. Since HLAs were reported to have strong homology to class II SLAs^53^, they were also used in MHCII binding predictions by other swine vaccine design studies^28,32,38^. Aside from the high binding affinity to MHCII molecules and high recognition potential to T-cell receptors, some of the final HTL epitopes also showed cytokine-inducing potential, particularly IFN-$\gamma$, IL-4, and IL-10. IFN-$\gamma$ can regulate ASFV replication by stimulating natural killer cells and macrophages to combat viral infections^26^; whereas IL-4 and IL-10, as anti-inflammatory cytokines, play a role in promoting immunoregulatory responses, thus, contributing to a balanced and safe vaccine profile.

All the predicted epitopes were joined using peptide linkers and an immunogenic adjuvant at the N-terminus. As observed in the study, the addition of 50S ribosomal protein L7/L12 as an adjuvant resulted in favorable solubility and stability of the vaccine construct. This adjuvant has been previously employed in several vaccine design studies^54–56^. It influences the maturation of dendritic cells (DCs), the most potent antigen-presenting cell, and the production of pro-inflammatory cytokines, which is partially mediated through the Toll-like receptor 4 (TLR4) signaling pathway^57^. The activation of DCs subsequently stimulates naïve T cells, leading to the effective polarization of CD4+ and CD8+ T cells, resulting in the secretion of IFN-$\gamma$ and initiation of T cell-mediated cytotoxic responses.

After designing the ASFV vaccine construct, further assessments of its physicochemical characteristics were conducted. While the assessment of epitopes is a critical aspect of vaccine development, focusing solely on epitopes overlooks important factors that contribute to the overall effectiveness and safety of a vaccine. The interaction between the components of the vaccine construct can significantly affect immune response. Therefore, understanding the effect of these components as a whole is crucial for designing a successful vaccine.

Overall, the designed ASFV vaccine has 364 amino acids and a molecular weight of 41 kDa. Proteins with a molecular weight under 110 kDa are often considered promising candidates for vaccine development^58^ owing to their solubility and the ease of purification^59^. The theoretical isoelectric point (pI) was predicted to be 7.22, aligned with the normal range of pH conditions^58^. Designing vaccines with a pI that matches physiological conditions increases the likelihood of maintaining protein solubility, preventing aggregation, and ensuring proper delivery and administration^60^. Furthermore, the SolPro server predicted that the vaccine construct is soluble at a high level of certainty (94%). The ProtParam server also predicted that the vaccine construct is stable. Moreover, it has an aliphatic index of 81.14, higher than the indices of vaccine constructs in other studies^61,62^. This high aliphatic content suggests substantial thermostability. Maintaining structural integrity at various temperatures is crucial for ensuring the effectiveness of vaccines during storage, transportation, and administration^63^. Aside from being antigenic, the ASFV vaccine construct was predicted to be non-allergenic and non-cross-reactive to swine populations, therefore it does not induce unintended immune reactions which ensures the safety of the designed vaccine.

A well-designed secondary structure of a vaccine enhances its immunogenicity and ensures effective immune response generation. Globular regions within the vaccine construct, with a substantial presence of $\alpha$-helices, suggest stable and well-structured regions in the vaccine. These structured regions are important in facilitating straightforward interactions with immune cells, thereby promoting a predictable immune response. While disordered regions can reduce the affinity of vaccine components to immune receptors^64^, they can serve as flexible regions that are capable of adapting to different conformations, enhancing the ability of the vaccine to engage with the complex landscape of the immune system.

Aside from the induction of adaptive immunity, the capability of inducing innate immune response was also tested by docking the ASFV vaccine construct with TLR4. Successful binding with Toll-like receptors (TLRs) streamlines vaccine-induced immune response, resulting in an improved ability to establish a robust memory for the target pathogen. Due to their ability to link innate with adaptive immune response, TLR-agonists are highly promising vaccine adjuvants^65^. Among 10 TLRs identified in swine^66^, TLR4 was used in this study for docking with the vaccine construct since the adjuvant of the vaccine, 50S ribosomal protein L7/L12 is a known TLR4-agonist. TLR4-agonists activate myeloid differentiation primary response protein 88 (MyD88) and Toll/IL-1R domain-containing adaptor inducing IFN-$\beta$ (TRIF) signaling pathways^67^, which may enhance activation of DCs and downstream immune responses, as described above.

Due to the absence of a specific swine TLR4 model, the study utilized the crystal structure of a human TLR4 (RCSB ID: 4G8A), which exhibits approximately 70% similarity to the swine counterpart^68^. This is the model closest to the structure of the swine TLR4 available in RCSB. For comparison, this study used three (3) TLR4 protein agonists: enzyme lumazine synthase from *Brucella* spp., resuscitation-promoting factor (Rpf) E from *Mycobacterium tuberculosis*, and fusion protein DnaJ-$\Delta$A146Ply from *Streptococcus pneumoniae*. In addition to the designed vaccine construct, docking analyses were also conducted with these three TLR4-agonists since no model or crystal structure is available in databases that show their binding and signaling mechanism with TLR4. The results of the docking of the ASFV vaccine construct to TLR4 revealed a highly negative $\Delta$G compared to controls, indicating a more favorable binding to the receptor. However, molecular dynamics simulation suggested a highly dynamic movement of the vaccine construct in complex with the TLR4. A relatively low eigenvalue compared to controls was observed, indicating that the complex with the ASFV vaccine construct was more flexible. This suggested that the vaccine can adapt and interact with TLR4 in various ways, enabling the formation of stable complexes via different binding modes.

Following optimization of the codon sequence of the vaccine construct, the codon adaptation index (CAI) reached a maximum possible value of 1.0. Additionally, 48.56% was the GC content of the optimized codon which is within the ideal range of 30-70%. Both parameters indicate optimal expression of the construct in the *E. coli* K-12 expression system.

Several proteins of ASFV have already shown promise in *in vivo* experiments. Majority of these are structural proteins, including p22 (KP177R), p30 (CP204L), p54 (E183L), p72 (B646L), and CD2v (EP402R)^19,23,69,70^. These proteins together with all proteins of ASFV were considered in the analysis. However, the results revealed that a greater number of epitopes from other protein sources exhibited higher immunogenicity than epitopes found on these structural proteins. Four out of the ten protein sources of the T-cell epitopes in the study, including polyprotein pp220 (CP2475L)^27,71,72^, guanylyltransferase (NP868R)^22^, helicase/primase (F1055L)^72^, and MGF 360-11L^20,72^, induced T-cell responses against ASFV in *in vitro* experiments. Meanwhile, all four protein sources of the LBL epitopes—minor capsid protein p49 (B438L)^73^, envelope protein p22^22,74^, transmembrane protein (C257)^22^, and major capsid protein p72 (B646L)^22,73–76^ have shown positive antibody response against the virus in *in vitro* studies. The six remaining T-cell epitope protein sources, including transmembrane (C62L) and five nonstructural proteins - cysteine protease (S273R), MGF 360-13L, MGF 360-18R, EP424R, and termination factor (Q706L) have not undergone extensive studies. Thus, further investigations are warranted to explore the ability of these proteins to induce an immune response.

Overall, the vaccine construct displayed favorable physicochemical properties and immunogenicity after a series of sequence and structure-based evaluations. By employing immunoinformatics, a safe, structurally stable, and immunogenic vaccine construct was made. This vaccine incorporates immunogenic epitope components of ASFV, emulating ASFV infection, and inducing immune memory to effectively counter subsequent infections of the pathogen. While immunoinformatics offers an efficient approach to vaccine development, it remains crucial to prioritize the conduct of updated benchmark studies across server platforms to select the most effective tools for specific applications and reduce the likelihood of suboptimal vaccine candidates being pursued. This vaccine design study incorporated the most updated benchmark studies to ensure that the vaccine construct remained aligned with the current studies and discoveries in immunology.

To optimize the design of multi-epitope subunit vaccines for swine, the establishment of dedicated prediction and evaluation servers specifically tailored for swine should also be prioritized. General prediction and evaluation servers may not provide accurate results for swine due to the differences in the immune system and genetics of hosts. It is important to note that all findings of the analysis were based on computational models of the servers used. *In vivo* investigations are necessary to validate the efficacy of the designed vaccine for ASFV.

## Methods

### Proteome screening and protein prioritization

All available ASFV proteomes were retrieved from UniProt^77^ (https://www.uniprot.org) and NCBI^78^ (https://www.ncbi.nlm.nih.gov) on 20 February 2023. CD-HIT^79^ (https://sites.google.com/view/cd-hit) was used to generate single representations of proteins with >80% sequence similarity and >75% alignment coverage.

Proteins from genotypes I and II were prioritized for screening of potential vaccine components. This was performed by first determining the genotypes of the isolates. Genotypes of ASFV isolates were identified using phylogenetic-based clustering analysis^80^. All ASFV genomes were retrieved from the NCBI database and aligned through MAFFT v7^81^ (https://mafft.cbrc.jp/alignment/server/) using default settings. With the same server, a neighbor-joining (NJ) tree of the conserved sites was constructed under the JTR model with 1000 bootstrapping replications. Identification of genotypes was completed through clade clustering using isolates with NCBI genotype assignments as references. To prioritize genotypes responsible for the recent global ASFV outbreaks, only proteins found in $\ge$80% of genotype I or $\ge$80% of genotype II isolates were selected.

Clustered sequences were aligned using Clustal Omega^82^ (https://www.ebi.ac.uk/Tools/msa/clustalo) and uploaded to the Protein Variability Server (PVS)^83^ (http://imed.med.ucm.es/PVS) to identify highly conserved fragments using Shannon variability entropy (H) of >1.0. Variability-masked sequences were kept for linear B-lymphocyte (LBL) epitope prediction. Contiguous conserved residues forming fragments of $\ge$9 were kept for cytotoxic T-lymphocyte (CTL) epitope prediction while contiguous conserved residues forming fragments of $\ge$15 were kept for helper T-lymphocyte (HTL) epitope prediction. These residues were selected based on the preference of Class I major histocompatibility complex (MHC) receptors for binding to 9-residue CTL epitopes^84^ and Class II MHC molecules for binding to 15-residue HTL epitopes^85^.

### Epitope prediction

BepiPred 3.0^86^ (https://services.healthtech.dtu.dk/services/BepiPred-3.0), SVMTrip^87^ (http://sysbio.unl.edu/SVMTriP/prediction. php), ABCPred^88^ (https://webs.iiitd.edu.in/raghava/abcpred), and LBTope^89^ (https://webs.iiitd.edu.in/raghava/lbtope) were used to predict Linear B-lymphocyte (LBL) epitopes. A threshold of 0.85 was used for ABCPred while parameters from other servers were kept at default. LBL epitopes were selected based on the following criteria: (1) presence in the variability-masked sequence, indicating conservation; (2) recognition as epitope by BepiPred; (3) recognition as epitope by either SVMTrip or ABCPred; (4) a $\ge$60% probability of correct prediction by LBTope; and (5) a length of $\ge$6 amino acids, as this is the minimum length accepted by antigenicity prediction.

A binding prediction server was first considered for screening CTL epitopes. The Immune Epitope Database Analysis (IEDB) MHCI binding prediction^90^ (http://tools.iedb.org/mhci) was used to select CTL epitopes with percentile rank (PR) $\le$0.5 for strong binders. Promiscuous epitopes were prioritized by selecting epitopes that strongly bind to 20% of the MHC molecules used^91^. Two processing servers were also used: IEDB MHCI processing^92^ (http://tools.iedb.org/processing) and NetCTLpan 1.1^93^ (https://services.healthtech.dtu.dk/services/NetCTLpan-1.1). IC50 concentration $\le$100 nM and proteasomal cleavage and TAP transport scores $\ge$1.0 were used in IEDB processing while PR $\le$0.5 in NetCTLpan 1.1.

Two servers were used for the selection of HTL epitopes: the IEDB MHCII binding prediction^94^ (http://tools.iedb.org/mhcii) with PR $\le$10 and NetMHCIIpan 4.1^95^ (https://services.healthtech.dtu.dk/services/NetMHCIIpan-4.0) with PR $\le$1.0. Promiscuous epitopes, as defined above, were also considered in HTL epitope prediction.

Several criteria were applied to select final LBL, CTL, and HTL epitopes: (1) antigenicity score $\ge$1.0 in Vaxijen 2.0^96^ (http://www.ddg-pharmfac.net/vaxijen/); (2) non-allergen in AllerTOP v2.0^97^ (https://www.ddg-pharmfac.net/AllerTOP); (3) non-toxic in ToxinPred^98^ (https://webs.iiitd.edu.in/raghava/toxinpred); (4) immunogenicity score >0.25 in the IEDB immunogenicity prediction^99^ (http://tools.iedb.org/immunogenicity), for CTL epitopes, while combined score <50 in the IEDB CD4 T-cell immunogenicity prediction^100^ (http://tools.iedb.org/CD4episcore) for HTL epitopes. IFNepitope^85^ (http://crdd.osdd.net/raghava/ifnepitope), IL4pred^101^ (http://webs.iiitd.edu.in/raghava/il4pred), and IL10pred^102^ (http://webs.iiitd.edu.in/raghava/ il10pred) were also used to determine the potential of HTL epitopes to induce cytokines.

### Vaccine construction and evaluation

Epitopes were joined together using “KK”, “AAY”, and “GPGPG” linkers while the “HEYGAEALERAG” linker was used to join the epitope groups. The linked epitopes were separately joined to five different adjuvants: *Sus scrofa* $\beta$-defensin-1^103^, F3-A6 ASFV hemagglutinin peptides^28^, phenol-soluble modulin $\alpha$4^104^, 50S ribosomal protein L7/L12^105^, and heparin-binding hemagglutinin adhesin (HBHA)^106^ using “EAAAK” linker.

Several criteria were also applied to select the final ASFV vaccine construct: (1) antigenicity score $\ge$0.40 in VaxiJen v2.0; (2) non-allergen in AllerTOP v2.0; (3) no homology (80% sequence similarity) to proteins of *Sus scrofa*, *S. scrofa domestica*, and *S. scrofa domesticus* in BlastP^107^ (https://blast.ncbi.nlm.nih.gov/Blast.cgi); (4) soluble upon overexpression in *Escherichia coli* using SCRATCH SolPro^108^ (https://scratch.proteomics.ics.uci.edu); and (5) stable in Expasy Protparam^109^ (https://web.expasy.org/protparam).

The secondary structure composition of the ASFV vaccine construct was determined using GOR4^110^ (https://npsa-prabi.ibcp.fr/ NPSA/npsa_gor4.html) while the globular and disordered regions were determined using GlobPlot 2.3^111^ (http://globplot.embl.de/). The tertiary structure was predicted with ColabFold v1.5.2-patch^112^ available in AlphaFold v.2.^113^. Templates were extracted in the pdb70 database where the top-ranking structure, with the highest average per-residue local distance difference test scores (pLDDT), was relaxed to improve local geometry. The predicted structure was refined through GalaxyRefine^114^ (https://galaxy.seoklab.org/cgi-bin/submit.cgi?type=REFINE) and visualized through ChimeraX^115,116^ (https://www.cgl.ucsf.edu/ chimerax/). The quality of the predicted structure was validated by Protein Structural Analysis (ProSA)^117^ (https://prosa.services.camesbg.ac.at/prosa.php) and SAVES v6.0 (https://saves.mbi.ucla.edu) ERRAT^118^ and PROCHECK^119^. The presence of conformational B-lymphocyte epitopes in the ASFV vaccine construct was predicted using the Discotope server^120^ (http://tools.iedb.org/discotope).

### Immune simulation

C-Immsim^121^ (https://kraken.iac.rm.cnr.it/C-IMMSIM/) was used to estimate the host immune response upon administration of the ASFV vaccine construct. Three (3) injections of 1000 vaccine particles were administered at 1–84–168 time-steps and the simulation was run until 300 time-step. The adjuvant used in the final vaccine construct was used as the control and was administered using the same parameters applied to the vaccine construct. The graphs of immune responses generated by the ASFV vaccine construct and the control were overlayed for comparison.

### Molecular docking and molecular dynamics

The study conducted two docking analyses: CTL epitopes to SLA1*0401 (from ID: 3QQ3^51^) and the designed ASFV vaccine to TLR4 (ID: 4G8A^122^). The crystal structures of the receptors were retrieved from RCSB^123^ (https://www.rcsb.org) and were cleaned using Pymol v2 (https://pymol.org/2), removing bound peptides and water molecules. CTL epitopes were docked into SLA1*0401 using GalaxyPepDock^124^ (https://galaxy.seoklab.org/cgi-bin/submit.cgi?type=PEPDOCK) while the designed vaccine was docked to the TLR4 using ClusPro 2.0^125^ (https://cluspro.bu.edu/login.php). Binding free energies ($\Delta$G) were evaluated using PRODIGY^126^ (https://wenmr.science.uu.nl/prodigy/) while molecular interactions in the complexes were predicted by PDBSum^127^ (http://www.ebi.ac.uk/thornton-srv/databases/pdbsum/). The influenza-derived epitope bound in SLA1*0401 in RCSB ID: 3QQ3 and another ebola-derived epitope also bound in SLA1*0401 in another crystal structure, RCSB ID: 3QQ4^51^, were used to compare the binding affinity of the final CTL epitopes to SLA1*0401 through their $\Delta$G values and nature of molecular interactions. Since the crystal structure of the obtained TLR4 did not initially include any docked molecule, three (3) known TLR4-agonists: enzyme lumazine synthase from *Brucella* spp.^128^, resuscitation-promoting factor (Rpf) E from *Mycobacterium tuberculosis*^129^, and fusion protein DnaJ-$\Delta$A146Ply from *Streptococcus pneumoniae*^130^ were docked to the TLR4 crystal structure to serve as control. The $\Delta$G values and molecular interactions of the TLR4-agonists complexes were compared to the ASFV vaccine construct. The stability of the TLR4 complexes was analyzed through the iMODS^131^ (https://imods.iqfr.csic.es/) web server.

### Codon optimization and in silico cloning

Java Codon Adaptation Tool (JCAT)^132^ (http://www.jcat.de/Start.jsp) was used to generate an optimized codon for the ASFV vaccine construct using the *Escherichia coli* K-12 expression system, avoiding rho-independent transcription terminators and prokaryotic ribosome binding sites. The restriction cloning module of the SnapGene tool (http://www.snapgene.com) was employed to clone the adapted nucleotide sequence into the pET-28a(+) vector with XhoI and BamHI restriction enzymes added at the N-terminal and C-terminal sites, respectively.

### Supplementary Information

Supplementary Information.

## Data Availability

All datasets on which the conclusions of the manuscript rely are presented in the paper. The raw data is available from the corresponding author (F.L.O.) upon reasonable request.
