# Supplementary material for: Immunoinformatics-guided approach for designing a pan-proteome multi-epitope subunit vaccine against African swine fever virus
Source: Sci Rep. 2024 Jan 16;14:1354. doi: 10.1038/s41598-023-51005-3 (PMC10791618; doi:10.1038/s41598-023-51005-3)
Supplement: Supplementary file 1 — Supplementary Information. [file 41598_2023_51005_MOESM1_ESM.pdf]

SUPPLEMENTARY INFORMATION

Immunoinformatics-guided Approach for Designing a Pan-Proteome Multi-Epitope Subunit Vaccine against African Swine Fever Virus

Alea Maurice Simbulan[1+], Edward C. Banico[1+], Ella Mae Joy S. Sira[1], Nyzar Mabeth O. Odchimar[1], Fredmoore L. Orosco[1*]

[1]Virology and Vaccine Institute of the Philippines Program

Industrial Technology Development Institute

Department of Science and Technology

Bicutan, Taguig City, Metro Manila, Philippines, 1634

[2]S&T Fellows Program

Department of Science and Technology

Bicutan, Taguig City, Metro Manila, Philippines, 1634

*corresponding author: orosco.fredmoore@gmail.com

[+]these authors contributed equally to this work

SUPPLEMENTARY TABLES

Supplementary Table 1: Metadata of the 100 proteomes included in the immunoinformatics-based designing of a multi-epitope subunit vaccine for African swine fever virus (ASFV).

No.	Strain Name (Year collected)	Source Database	Accession no.	UniProt ID	Host	Genotype
1	Kyiv (2016)	NCBI/UniProt	MN194591.1	UP000321214	Domestic pig	II
2	Sardinia Sassari (2008)	NCBI/UniProt	KX354450.1	UP000266411	Domestic pig	I
3	Italy (57/Ca) (1979)	NCBI/UniProt	MN270970.1	UP000500690	Domestic pig	I
4	Italy (56/Ca) (1978)	NCBI/UniProt	MN270969.1	UP000500898	Domestic pig	I
5	Italy (141/Nu) (1990)	NCBI/UniProt	MN270974.1	UP000501235	Domestic pig	I
6	Italy (26/Ss) (2004)	NCBI/UniProt	MN270977.1	UP000501683	Domestic pig	I
7	Italy (140/Or) (1985)	NCBI/UniProt	MN270972.1	UP000501990	Domestic pig	I
8	Italy (97/Ot) (2012)	NCBI/UniProt	MN270979.1	UP000502885	Domestic pig	I
9	Italy (139/Nu) (1981)	NCBI/UniProt	MN270971.1	UP000502933	Domestic pig	I
10	Italy (142/Nu) (1995)	NCBI/UniProt	MN270975.1	UP000503294	Domestic pig	I
11	Italy (60/Nu) (1997)	NCBI/UniProt	MN270976.1	UP000501465	Domestic pig	I
12	Italy (85/Ca) (1985)	NCBI/UniProt	MN270973.1	UP000501487	Domestic pig	I
13	Italy (22653/Ca) (2014)	NCBI/UniProt	MN270980.1	UP000502695	Domestic pig	I
14	Italy (72407/Ss) (2005)	NCBI/UniProt	MN270978.1	UP000503066	Wild boar	I
15	France (Liv13/33) (OmLF2) (2017)	NCBI/UniProt	MN913970.1	UP000500872	Tick	I
16	Italy (103917) (2018)	NCBI/UniProt	MT932578.1	UP000594565	Domestic pig	I
17	Italy (55234) (2018)	NCBI/UniProt	MT932579.1	UP000594644	Domestic pig	I
18	South Korea (Yeoncheon1) (2019)	NCBI	MW049116.1	N/A	Domestic Pig	II
19	Philippines (A4) (2021)	NCBI	ON963982.1	N/A	Wild boar	II
20	Italy (20355/RM) (2022)	NCBI	OP605386.1	N/A	Wild boar	II
21	Belgium (1) (2018)	UniProt	LR536725.1	UP000307568	Domestic pig	II
22	Czech Republic (1) (2017)	UniProt	LR722600.1	UP000327056	Wild boar	II
23	Georgia (1) (2007)	NCBI/UniProt	FR682468.2	UP000141072	Domestic pig	II
24	Moldova (Moldova/1) (2017)	UniProt	LR722599.1	UP000325567	Domestic pig	II
25	China (GZ201801_2) (2018)	NCBI	ON263123.1	N/A	Domestic Pig	II
26	Hungary (2018)	NCBI/UniProt	MN716334.1	UP000428265	Wild boar	II
27	Zambia (LIV 5/40) (1983)	NCBI/UniProt	MN318203.3	UP000427047	Tick	I
28	Lithuania (LT14/1490) (2014)	NCBI/UniProt	MK628478.1	UP000326051	Wild boar	II
29	South Korea (YC1) (2019)	NCBI	ON075797.1	N/A	Wild boar	II
30	Poland (28298_O111) (2018)	NCBI/UniProt	MT847621.1	UP000593631	Wild boar	II
31	Poland (55892_C754) (2017)	NCBI/UniProt	MT847620.1	UP000594088	Domestic pig	II
32	Poland (53050_C1959) (2019)	NCBI/UniProt	MT847623.2	UP000593778	Wild boar	II
33	Poland (31177_O81) (2017)	NCBI/UniProt	MT847622.1	UP000593931	Wild boar	II
34	Germany (1) (2020)	UniProt	LR899193.1	UP000594902	Domestic pig	II
35	China (HLJ/Pig) (2018)	NCBI/UniProt	MK333180.1	UP000291821	Domestic pig	II
36	China (DB/LN) (2018)	NCBI/UniProt	MK333181.1	UP000292678	Domestic pig	II
37	China (wbBS01) (2018)	NCBI/UniProt	MK645909.1	UP000316600	Wild boar	II
38	China (Pig/HRB1) (2020)	NCBI/UniProt	MW656282.1	UP000671820	Domestic pig	II
39	China (wbShX01) (2019)	NCBI	MW033528.1	N/A	wild boar	II
40	China (SY-1) (2020)	NCBI	OM161110.1	N/A	Wild boar	II
41	China (SY-2) (2021)	NCBI	OP612163.1	N/A	Bama mini-pig	II
42	Belgium (Etalle/wb) (2018)	NCBI/UniProt	MK543947.1	UP000324915	Wild boar	II
43	China (InnerMongolia-AES01) (2019)	NCBI/UniProt	MK940252.1	UP000595256	Wild boar	II
44	China (wild boar/SNJ) (2020)	NCBI	OL622042.1	N/A	Wild boar	II
45	China (CAS19-01) (2019)	NCBI/UniProt	MN172368.1	UP000422299	Domestic pig	II
46	Japan (AQS-C-1-21) (2021)	NCBI	LC659086.1	N/A	Swine	II
47	Japan (AQS-C-1-22) (2021)	NCBI	LC659087.1	N/A	Swine	II
48	Japan (AQS-P-20901-1) (2021)	NCBI	LC659088.1	N/A	Swine	II
49	Japan (AQS-P-201202) (2021)	NCBI	LC659089.1	N/A	Swine	II
50	Russia (Kaliningrad/WB-10168) (2019)	NCBI	OM966719.1	N/A	Wild boar	II
51	South Korea (HC224) (2020)	NCBI	OP628183.1	N/A	Wild boar	II
52	Russia (Kabardino-Balkaria/WB-964) (2019)	NCBI/UniProt	MT459800.1	UP000594604	Wild boar	II
53	Russia (Kaliningrad/WB-12523) (2018)	NCBI	OM966714.1	N/A	Wild boar	II
54	Russia (Kaliningrad/WB-12524) (2018)	NCBI	OM966715.1	N/A	Wild boar	II
55	Russia (Kaliningrad/WB-9735) (2018)	NCBI	OM966716.1	N/A	Wild boar	II
56	Russia (Kaliningrad/WB-9763) (2018)	NCBI	OM966717.1	N/A	Wild boar	II
57	Russia (Kaliningrad/WB-9766) (2018)	NCBI	OM966718.1	N/A	Wild boar	II
58	Russia (Kaliningrad/WB-12516) (2018)	NCBI	OM966720.1	N/A	Wild boar	II
59	Russia (Kaliningrad/WB-9734) (2018)	NCBI	OM966721.1	N/A	Wild boar	II
60	Russia (Primorsky/WB-6723) (2019)	NCBI/UniProt	MW306191.1	UP000675950	Wild boar	II
61	Russia (Ulyanovsk/WB-5699) (2019)	NCBI/UniProt	MW306192.1	UP000678137	Wild boar	II
62	China (AnhuiXCGQ) (2018)	NCBI/UniProt	MK128995.1	UP000290386	Domestic pig	II
63	China (GZ201801) (2018)	NCBI/UniProt	MT496893.1	UP000510925	Domestic pig	II
64	Timor-Leste (1) (2019)	NCBI/UniProt	MW396979.1	UP000664916	Domestic pig	II
65	Russia (Amur/WB-6905) (2019)	NCBI/UniProt	MW306190.1	UP000680325	Wild boar	II
66	Russia (Kaliningrad/WB-1386) (2017)	NCBI	OM799941.1	N/A	Wild boar	II
67	Kenya (Ken.rie1) (2019)	UniProt	LR899131.1	UP000594880	Tick	X

Supplementary Table 1: (continued)

No.	Strain Name (Year collected)	Source Database	Accession no.	UniProt ID	Host	Genotype
68	Uganda (R7) (2015)	NCBI/UniProt	MH025917.1	UP000276891	Domestic pig	IX
69	Uganda (R8) (2015)	NCBI/UniProt	MH025916.1	UP000275389	Domestic pig	IX
70	Uganda (R35) (2015)	NCBI/UniProt	MH025920.1	UP000282163	Domestic pig	IX
71	Vietnam (NgheAn) (2019)	NCBI/UniProt	MT180393.1	UP000595502	swine	II
72	China (LYG18) (2018)	NCBI	OM105586.1	N/A	Wild boar	II
73	Uganda (R25) (2015)	NCBI/UniProt	MH025918.1	UP000273742	Domestic pig	IX
74	South Africa (RSA/2 (2008)	NCBI/UniProt	MN336500.3	UP000422855	Tick	XXII
75	Uganda (N10) (2015)	NCBI/UniProt	MH025919.1	UP000267661	Domestic pig	IX
76	Kenya (Tk1) (2005)	NCBI/UniProt	KM111294.1	UP000105860	Tick	X
77	South Africa (SPEC 57) (1985)	NCBI/UniProt	MN394630.3	UP000423628	Tick	VIII
78	South Africa (RSA_2) (2004)	NCBI/UniProt	MN641877.2	UP000502315	Wild boar	XX
79	South Africa (RSA_W1) (1999)	NCBI/UniProt	MN641876.2	UP000503015	Warthog	IV
80	Congo (Zaire) (1977)	NCBI/UniProt	MN630494.2	UP000501719	Wild boar	XX
81	China (JX21) (2021)	NCBI	OM105587.1	N/A	Wild boar	II
82	Dominican Republic (1980)	NCBI	ON185726.2	N/A	Wild boar	I
83	Italy (Sardinia/26544/OG10) (2010)	NCBI/UniProt	KM102979.1	UP000117635	Domestic pig	I
84	China (YNFN202103) (2021)	NCBI	ON400500.1	N/A	Domestic Pig	II
85	Portugal (Lisbon) (1960)	NCBI/UniProt	KM262844.1	UP000142390	Domestic pig	I
86	Spain (E75) (1975)	NCBI/UniProt	FN557520.1	UP000101090	Domestic pig	I
87	Estonia (2014)	NCBI/UniProt	LS478113.1	UP000267045	Wild boar	II
88	China (Wuhan-2) (2019)	NCBI/UniProt	MN393477.1	UP000502194	Domestic pig	II
89	China (Wuhan-1) (2019)	NCBI/UniProt	MN393476.1	UP000503567	Domestic pig	II
90	Kenya (Ken06.Bus) (2006)	NCBI/UniProt	KM111295.1	UP000101566	Domestic pig	IX
91	Spain (BA71V) (1971)	NCBI/UniProt	U18466.2	UP000000624	Tissue adapted	I
92	Namibia (Wart80) (1980)	NCBI/UniProt	AY261366.1	UP000000858	Warthog	IV
93	Portugal (NHV) (1968)	NCBI/UniProt	KM262845.1	UP000110401	Domestic pig	I
94	Vietnam (VNUA-05L1/HaNam/VN) (2020)	NCBI/UniProt	MW465755.1	UP000663149	Wild boar	II
95	Congo (Uvira B53) (2019)	NCBI/UniProt	MT956648.1	UP000663362	Domestic Pig	X
96	Kenya (1950)	NCBI/UniProt	AY261360.1	UP000000861	Domestic pig	X
97	Pretoriuskop (Pr4) (1996)	NCBI/UniProt	AY261363.1	UP000000859	Tick (Ornithodoros)	XX
98	Portugal (OURT88) (1988)	NCBI/UniProt	AM712240.1	UP000108903	Tick	I
99	Benin (1) (1997)	NCBI/UniProt	AM712239.1	UP000130745	Domestic pig	I
100	Malawi (Lil-20) (1983)	NCBI/UniProt	AY261361.1	UP000000860	Domestic pig	XIII

Supplementary Table 2: Physicochemical properties of the vaccine candidates for African swine fever virus (ASFV) upon addition of different adjuvants.

**Adjuvants**	**Antigenicity** (VaxiJen v2.0)	**Allergenicity** (AllerTOP v2.0)	**Cross-reactivity** (BlastP)	**Solubility** (SCRATCH SolPro)	**Stability** (Expasy ProtParam)
Sus scrofa β-defensin-1	Antigenic (0.8852)	Non-allergen	*Sus scrofa* β-defensin (100% identity)	Insoluble (at 73% probability)	Unstable (Instability =40.88)
F3-A6 hemagglutinin peptides	Antigenic (0.8652)	Non-allergen	None	Soluble (at 71% probability)	Unstable (Instability =40.84)
Phenol-soluble modulin α4	Antigenic (0.8398)	Non-allergen	None	Insoluble (at 72% probability)	Stable (Instability =37.31)
50S ribosomal protein L7/L12	Antigenic (0.7235)	Non-allergen	*Sus scrofa* L12 (35% identity)	Soluble (at 94% probability)	Stable (Instability =33.68)
Heparin-binding hemagglutinin adhesin	Antigenic (0.6523)	Non-allergen	None	Soluble (at 94% probability)	Unstable (Instability =43.79)

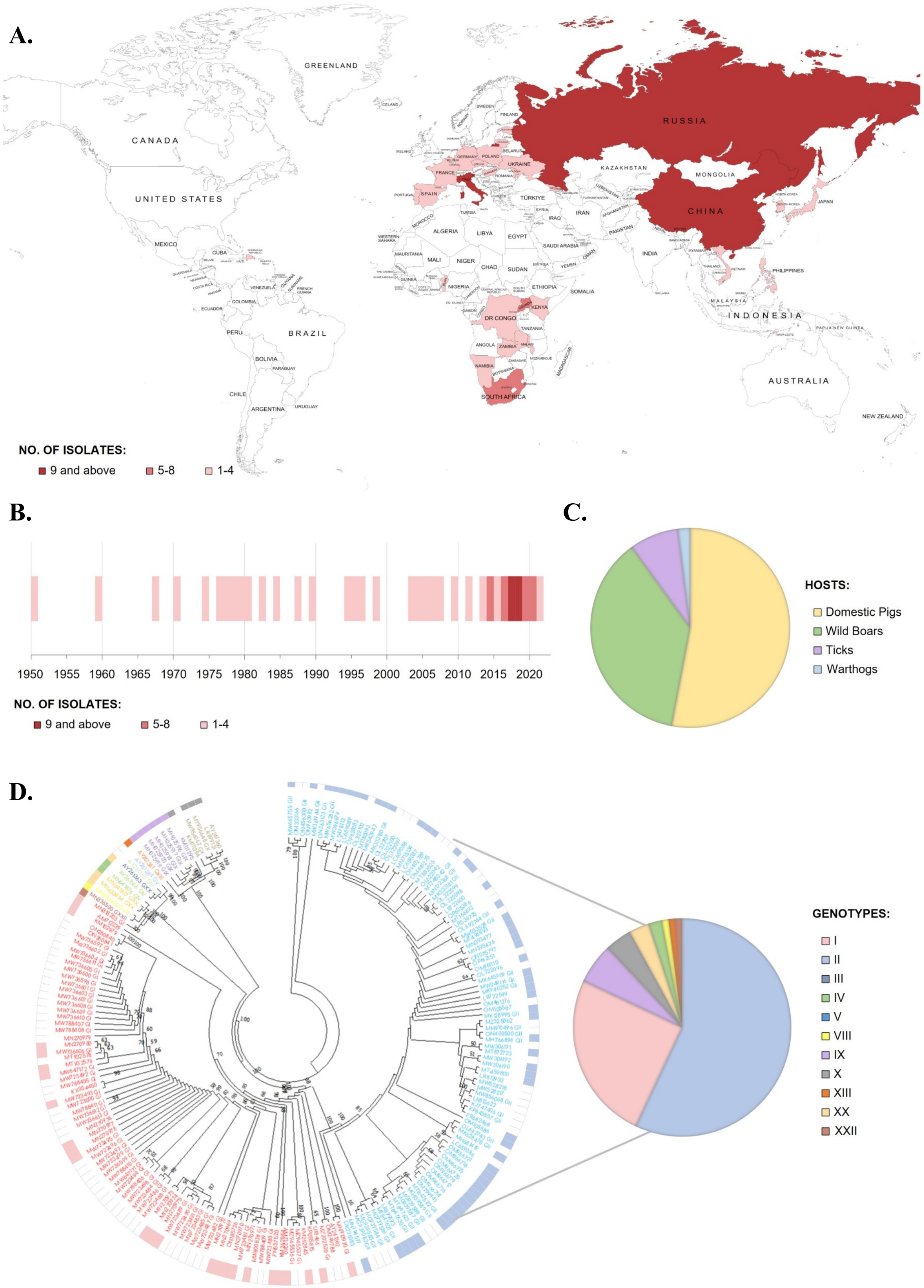

Supplementary Figure 1: Distribution of the 100 African swine fever virus (ASFV) isolates included in the immunoinformatics-based designing of a multi-epitope subunit vaccine for ASFV. (**A**) Geographical map marked with the countries from where the isolates were collected. (**B**) Year distribution from when the isolates were collected. (**C**) Distribution of the hosts from where the isolates were isolated. (**D**) Clustering analyses of 197 ASFV sequences through NJ tree algorithm (colored outer blocks represent the genotypes of isolates included in the study; tagged after accession numbers are genotypes of reference isolates).

SUPPLEMENTARY FIGURES

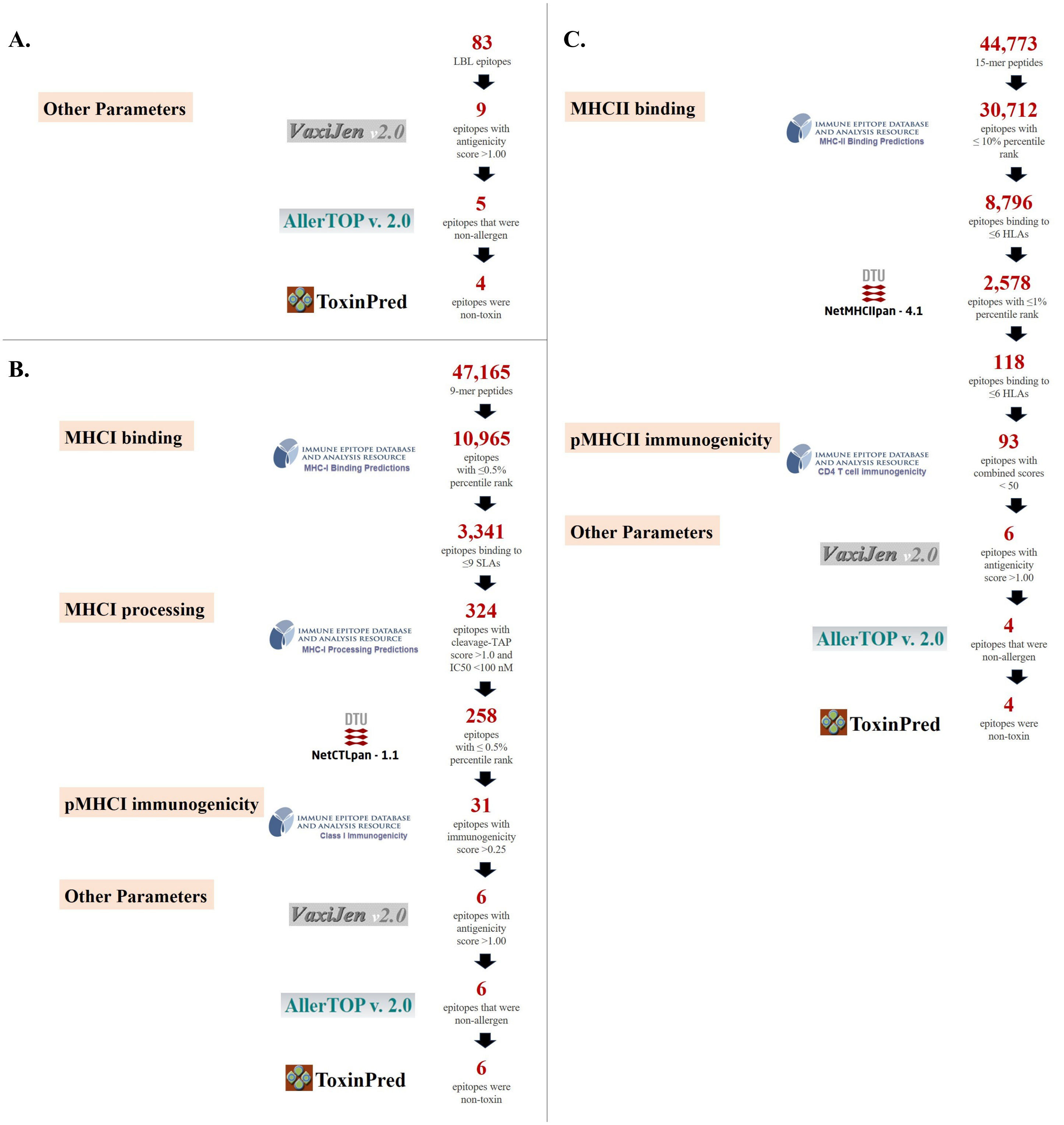

Supplementary Figure 2: Flowchart depicting the epitope prediction procedures followed to reach the selected epitopes in the study. (**A**) Screening process for the identification of the final linear B-lymphocyte (LBL) epitopes from the 83 putative LBL epitopes that were identified by BepiPred 3.0, SVMTrip, ABCPred, and LBTope. (**B**) Screening process for the identification of the final cytotoxic T-lymphocyte (CTL) epitopes from the 47,165 9-mer conserved peptides. (**C**) Screening process for the identification of the final helper T-lymphocyte (HTL) epitopes from the 44,473 15-mer conserved peptides.

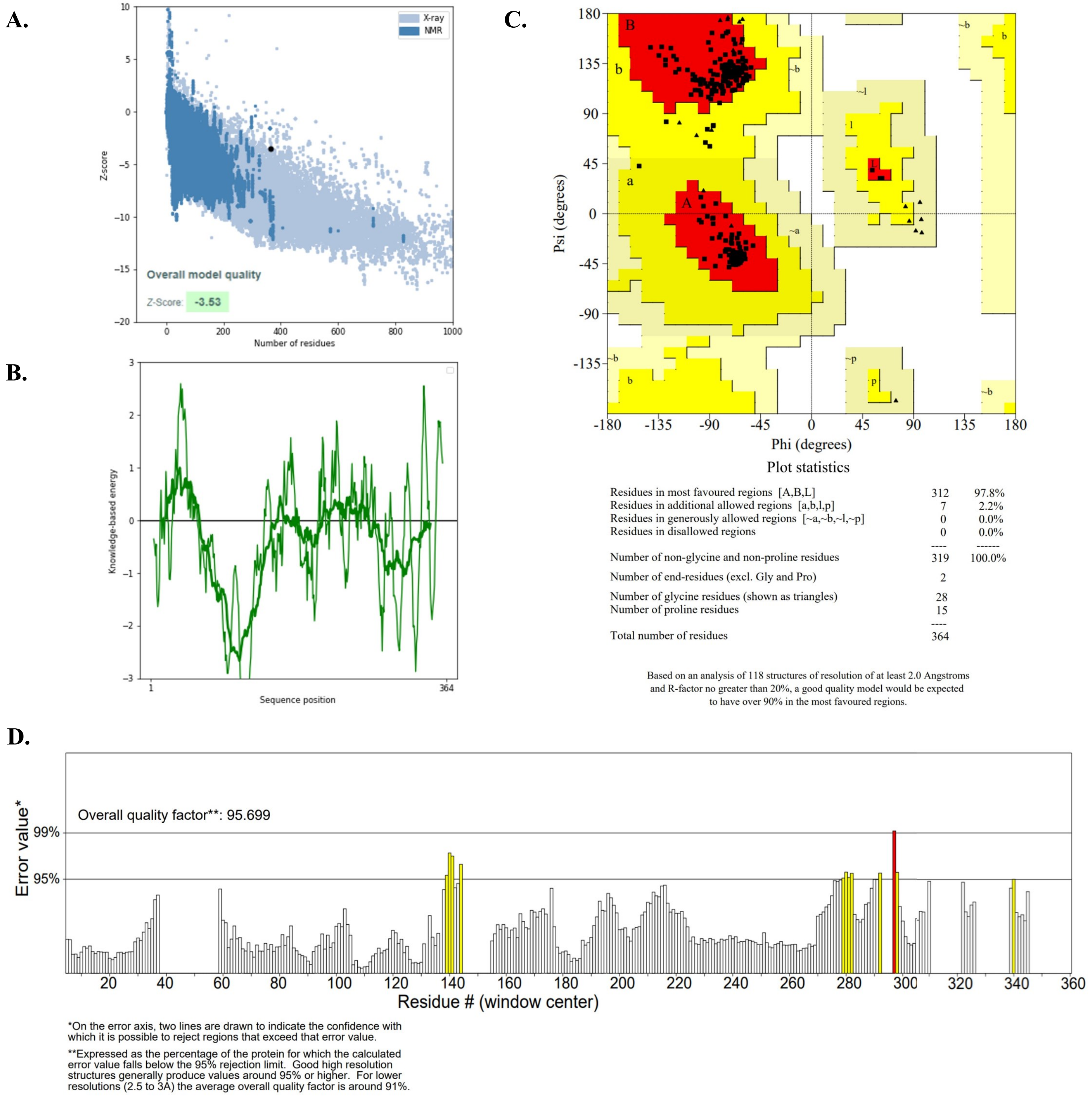

Supplementary Figure 3: Tertiary structure quality validation assessments of the African swine fever virus (ASFV) vaccine construct. (**A**) ProSA-web z-scores of all protein chains in PDB determined by X-ray crystallography (light blue) or NMR spectroscopy (dark blue) with respect to their length. The z-score of the ASFV vaccine construct is highlighted as large dot. (**B**) ProSA-web energy plot of the ASFV vaccine construct showing the local model quality by plotting energies as a function of amino acid sequence position. Positive values correspond to problematic or erroneous parts of the model. (**C**) Ramachandran plot generated by PROCHECK validation server showing the stereochemical quality of the tertiary structure of the ASFV vaccine construct. Dark red regions indicate most favored regions, dark yellow regions indicate additional allowed regions, and light-yellow regions indicate generously allowed regions. General amino acids are indicated in squares, glycine residues are indicated in triangles. (**D**) ERRAT plot shows error values for the residues within the ASFV vaccine construct.

A.

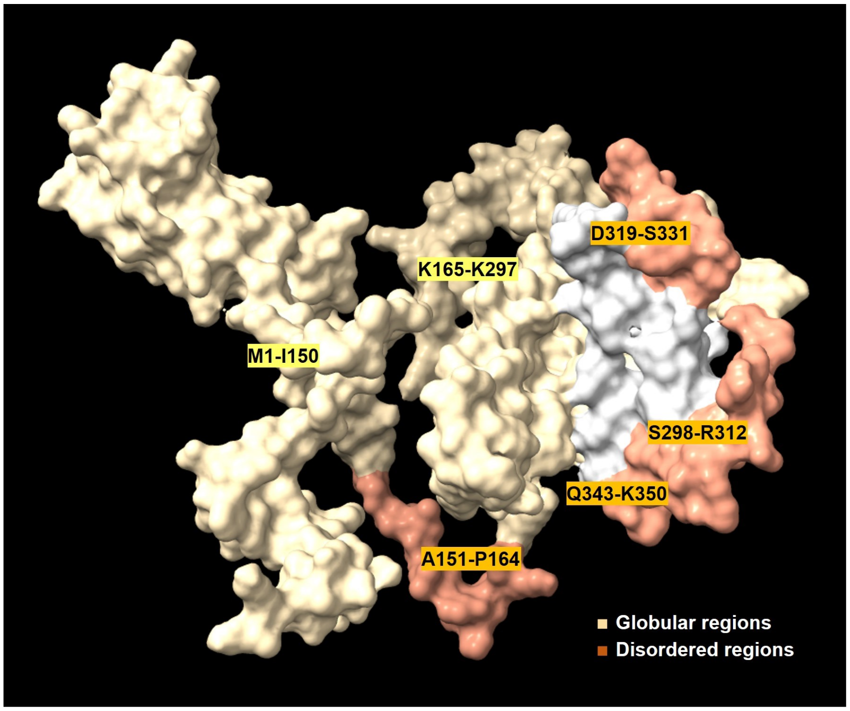

B.

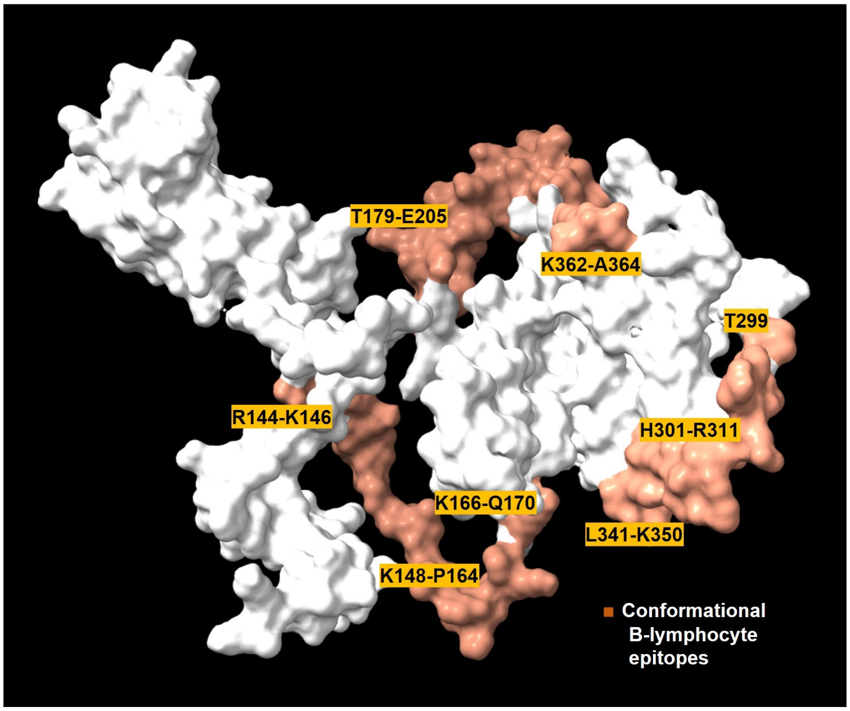

Supplementary Figure 4: Tertiary structure of the African swine fever virus (ASFV) vaccine construct highlighting globular-disordered regions and conformational B-lymphocyte epitope regions. (**A**) Globular and disordered regions identified in the ASFV vaccine construct using GlobPlot2. (**B**) Conformational B-lymphocyte epitope regions within the ASFV vaccine construct predicted by Discotope 1.1.

A.

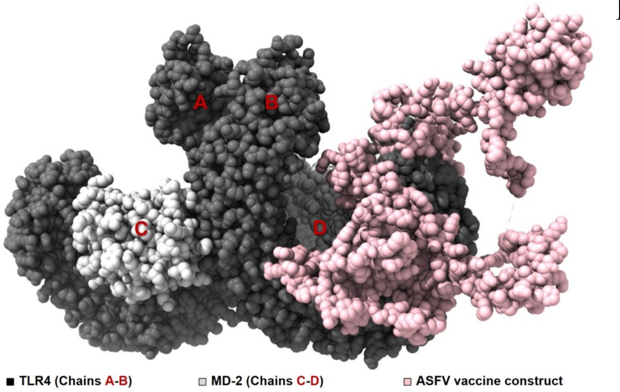

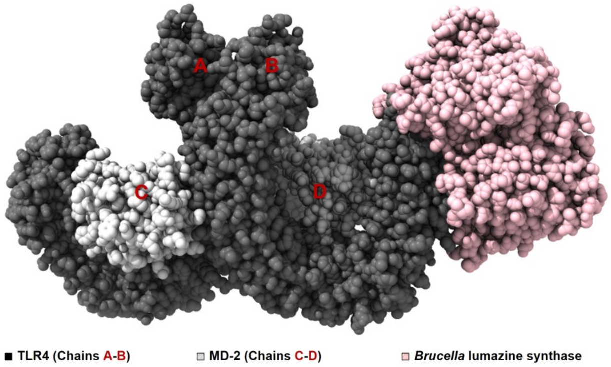

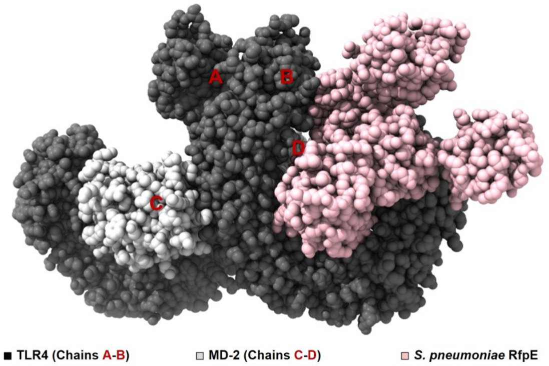

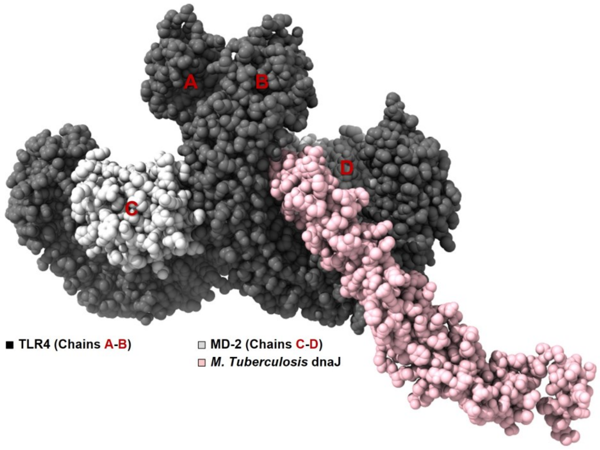

B.

Bonds:

TLR4					ASFV vaccine construct				
HYDROGEN BONDS									
Atom No.	Atom Name	Residue Name	Residue No.	Chain	Atom No.	Atom Name	Residue Name	Residue No.	Distance
4974	O	ASN	44	B	162	N	LEU	18	3.17
5096	ND2	ASN	58	B	2433	OD1	ASN	258	2.56
5289	NE2	GLN	81	B	2438	O	ASN	258	3.31
5643	NE2	GLN	129	B	2497	OE2	GLU	264	2.63
6015	O	ASN	176	B	2534	OH	TYR	268	3.05
6036	OE2	GLU	178	B	3322	OH	TYR	351	2.64
6448	NE2	HIS	229	B	3283	O	PRO	346	2.99
6655	ND1	HIS	256	B	3266	OE1	GLN	344	2.73

Bonds:

TLR4					*Brucella* lumazine synthase				
HYDROGEN BONDS									
Atom No.	Atom Name	Residue Name	Residue No.	Chain	Atom No.	Atom Name	Residue Name	Residue No.	Distance
5171	NH1	ARG	67	B	5402	O	HIS	120	3.02
SALT BRIDGES									
5032	OD1	ASP	50	B	5398	NE2	HIS	119	2.86
5393	OE1	GLU	94	B	3114	ND1	HIS	119	3.31
5402	OD2	ASP	95	B	3124	ND1	HIS	120	3.95
5401	OD1	ASP	95	B	3145	ND1	HIS	121A	2.98

Bonds:

TLR4					*S. pneumoniae* RfpE				
HYDROGEN BONDS									
Atom No.	Atom Name	Residue Name	Residue No.	Chain	Atom No.	Atom Name	Residue Name	Residue No.	Distance
4845	OE2	GLU	27	B	1103	N	ASN	100	3.02
4874	OE2	GLU	31	B	1975	NH2	ARG	146	3.16
4902	ND2	ASN	35	B	2517	OE2	GLU	147	1.84
5089	NZ	LYS	57	B	2878	OH	TYR	122	2.59
5272	OE1	GLU	79	B	2878	OH	TYR	122	2.98
5462	NE2	HIS	103	B	2842	O	GLY	118	2.67
7835	OH	TYR	403	B	3179	NH1	ARG	163	2.54
7835	OH	TYR	403	B	3180	NH2	ARG	163	2.42
7850	OD1	ASP	405	B	3179	NH1	ARG	163	2.75
8429	NZ	LYS	477	B	998	O	GLN	160	3.06
9414	NE	ARG	598	B	152	O	ASN	119	2.62
9425	NE2	GLN	599	B	2842	O	GLY	118	2.75
SALT BRIDGES									
4874	OE2	GLU	31	B	1975	NH2	ARG	146	3.16
7850	OD1	ASP	405	B	2646	NH1	ARG	163	2.75
8004	OE2	GLU	425	B	2698	NE	ARG	170	2.93
8030	OD2	ASP	428	B	2644	NE	ARG	163	3.34
11784	OE2	GLU	144	D	2073	NE	ARG	158	3.88

Bonds:

TLR4					*M. Tuberculosis* dnaJ				
HYDROGEN BONDS									
Atom No.	Atom Name	Residue Name	Residue No.	Chain	Atom No.	Atom Name	Residue Name	Residue No.	Distance
6011	OG1	THR	175	B	114	NE2	GLN	122	3.31
6430	NH2	ARG	227	B	1094	OH	TYR	253	2.81
8227	OH	TYR	451	B	509	ND2	ASN	175	2.53
8243	OD2	ASP	453	B	593	NH1	ARG	186	2.17
SALT BRIDGES									
6430	NH2	ARG	227	B	1074	OD2	ASP	251	3.75
8243	OD2	ASP	453	B	593	NH1	ARG	186	2.17
11775	OE2	GLU	143	D	604	NH1	ARG	187	3.12

Supplementary Figure 5: Molecular docking of the Toll-like receptor 4 (TLR4) to African swine fever virus (ASFV) vaccine construct and controls using ClusPro 2.0. (**A**) Tertiary structures of the proteins docked to the TLR4-myeloid differentiation-2 (MD-2) dimer complex. (**B**) Molecular bonds formed through the docking of the proteins to the TLR4-MD2 as predicted by PDBSum.

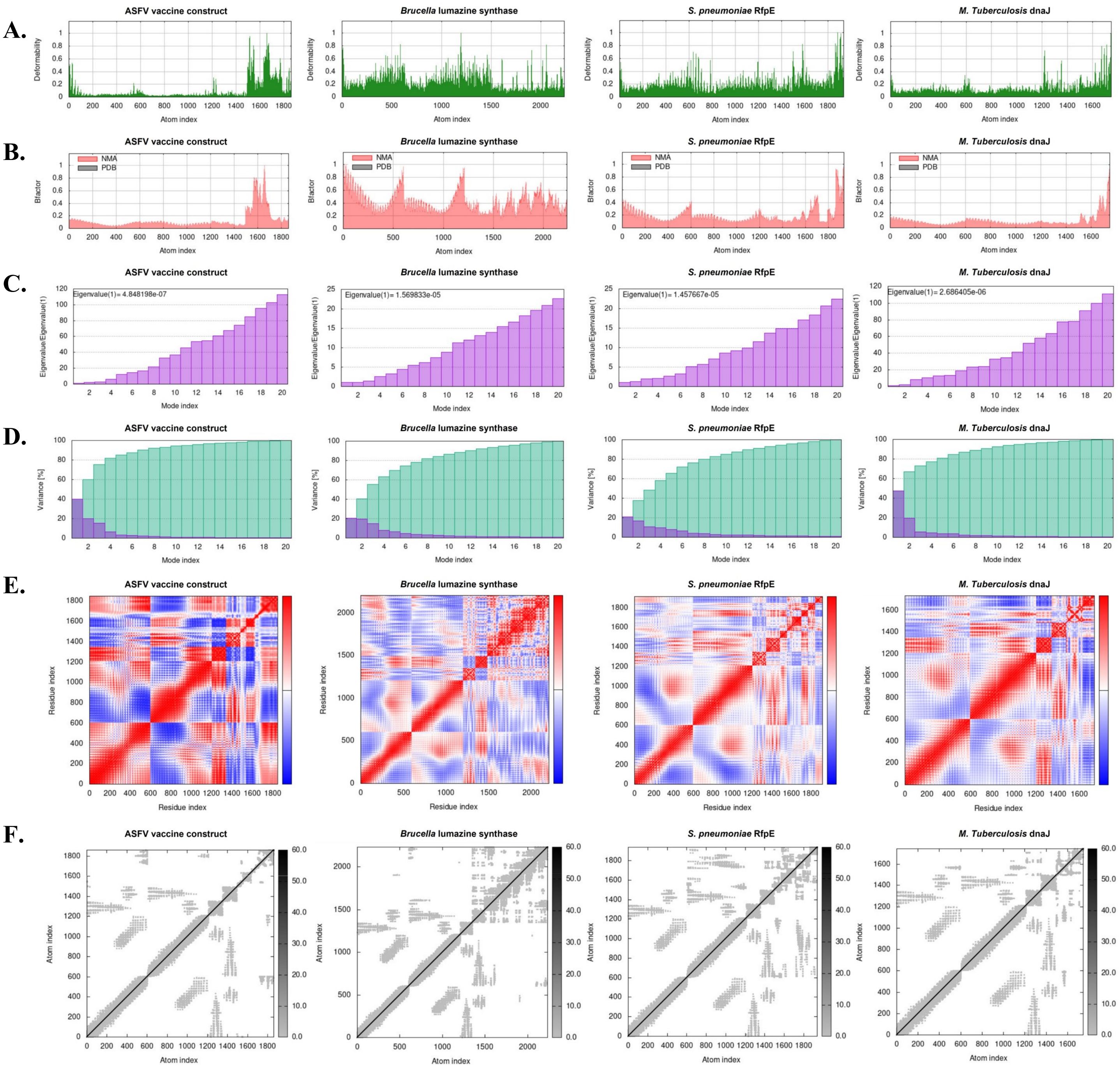

Supplementary Figure 6: Molecular dynamics simulation of the complexes formed by docking Toll-like receptor 4 (TLR4) to African swine fever virus (ASFV) vaccine construct and controls using iMODS. (**A**) Main-chain deformability graph generated by summing atomic displacements across all modes per residue highlights deformable protein regions. (**B**) NMA-derived B-factor graph indicating relative atomic displacement amplitudes around equilibrium. This illustrates the relationship between the mobility of the docked complex NMA and the PDB scores, representing average RMSDs. (**C**) Eigenvalues graph indicating motion stiffness for each mode index with lower values signifying easier alpha carbon deformations. (**D**) Variance graph showing individual and cumulative variances for each mode index, showing their contribution to the overall complex motion. (**E**) Covariance map highlighting correlated (red), uncorrelated (white), or anti-correlated (blue) atomic movements within the complex. (**F**) Elastic network model indicating atom relationships, with dot colors indicating stiffness; darker greys indicate stiffer regions, whereas lighter dots indicate flexible regions.
